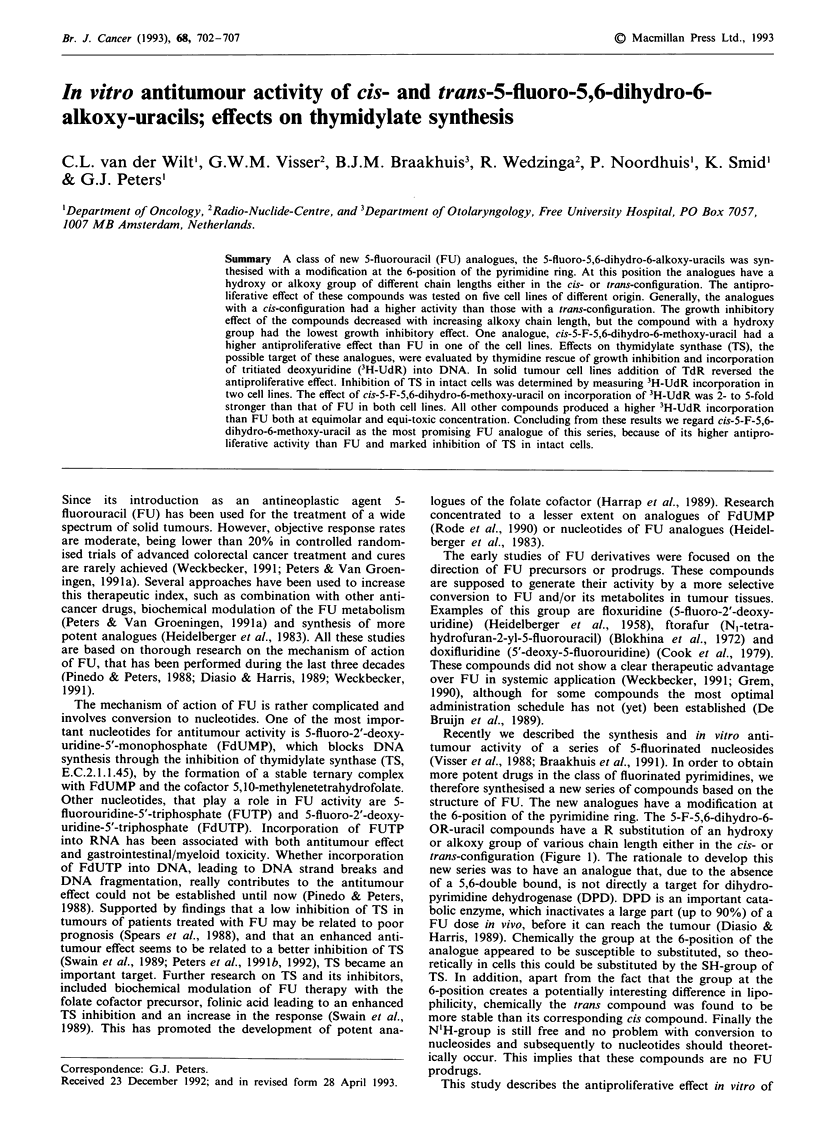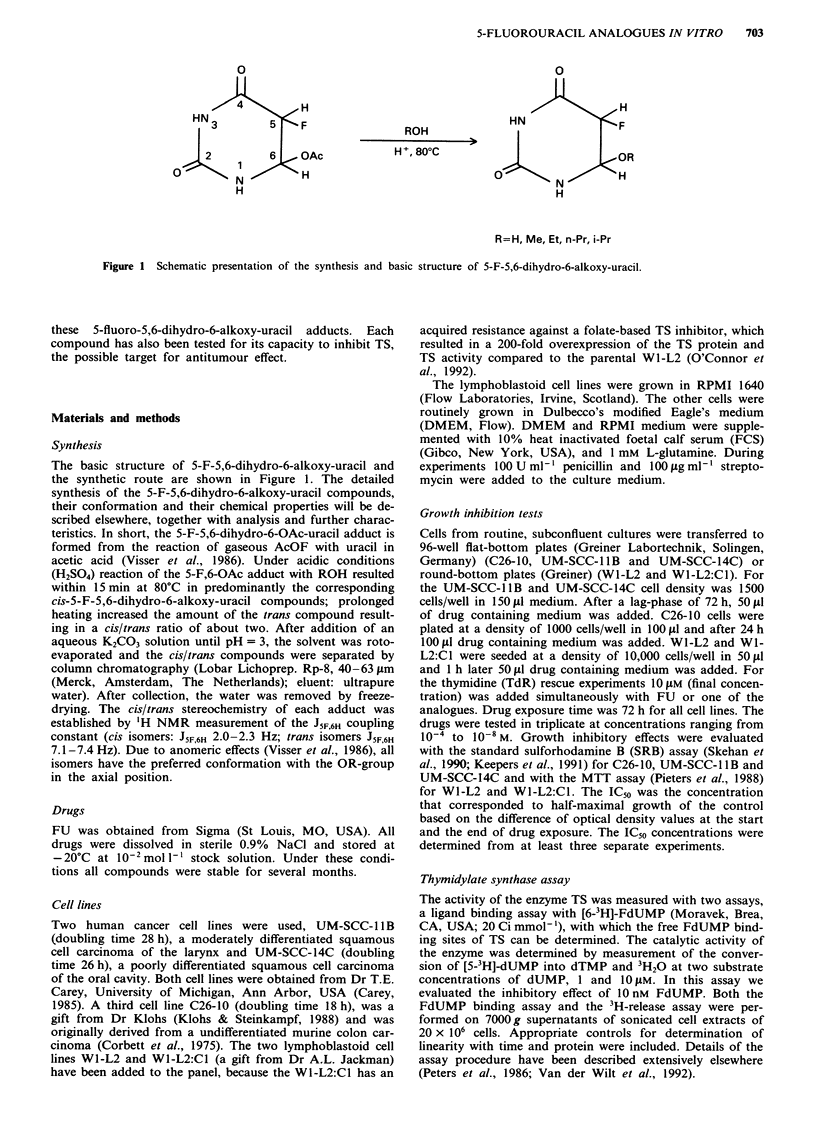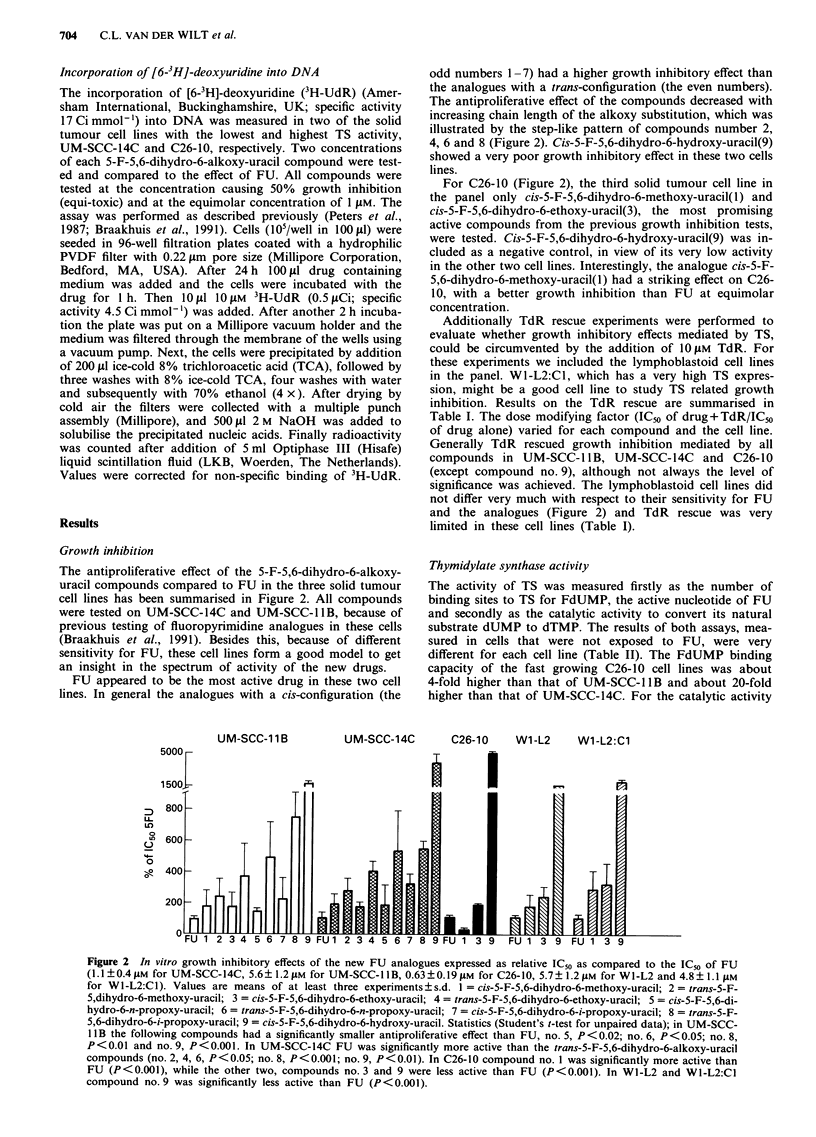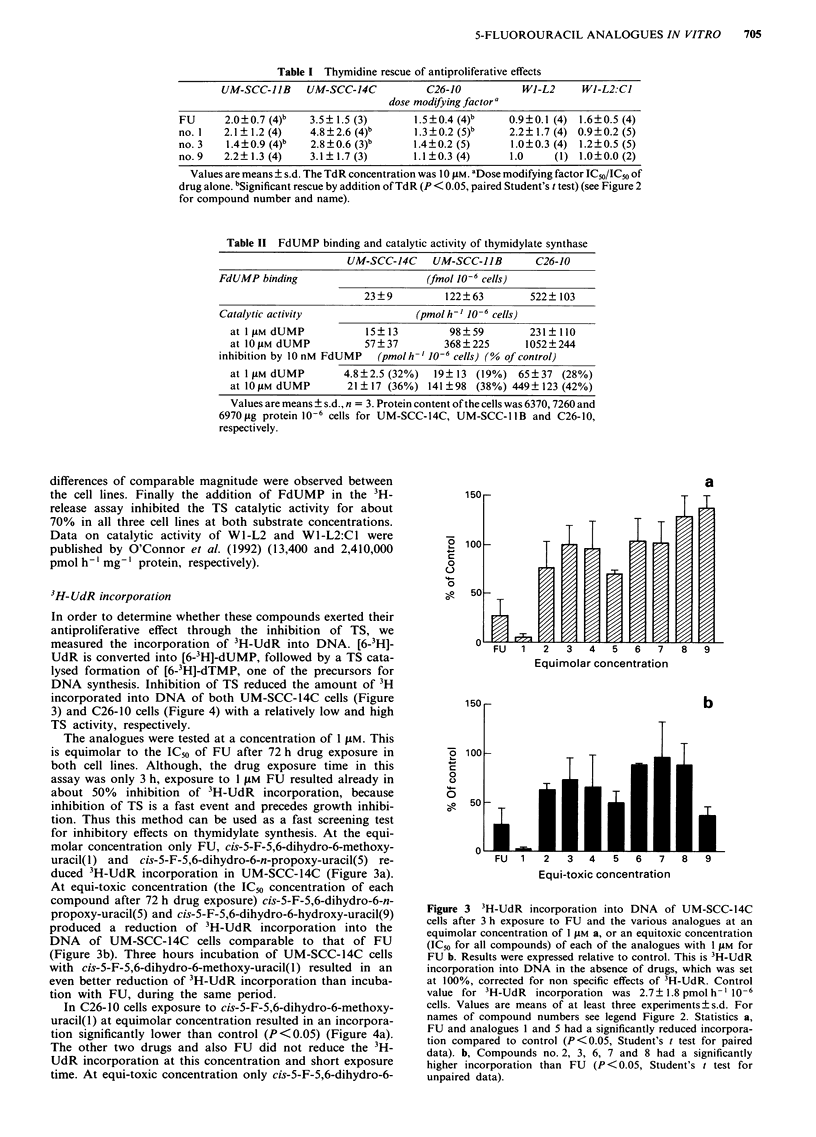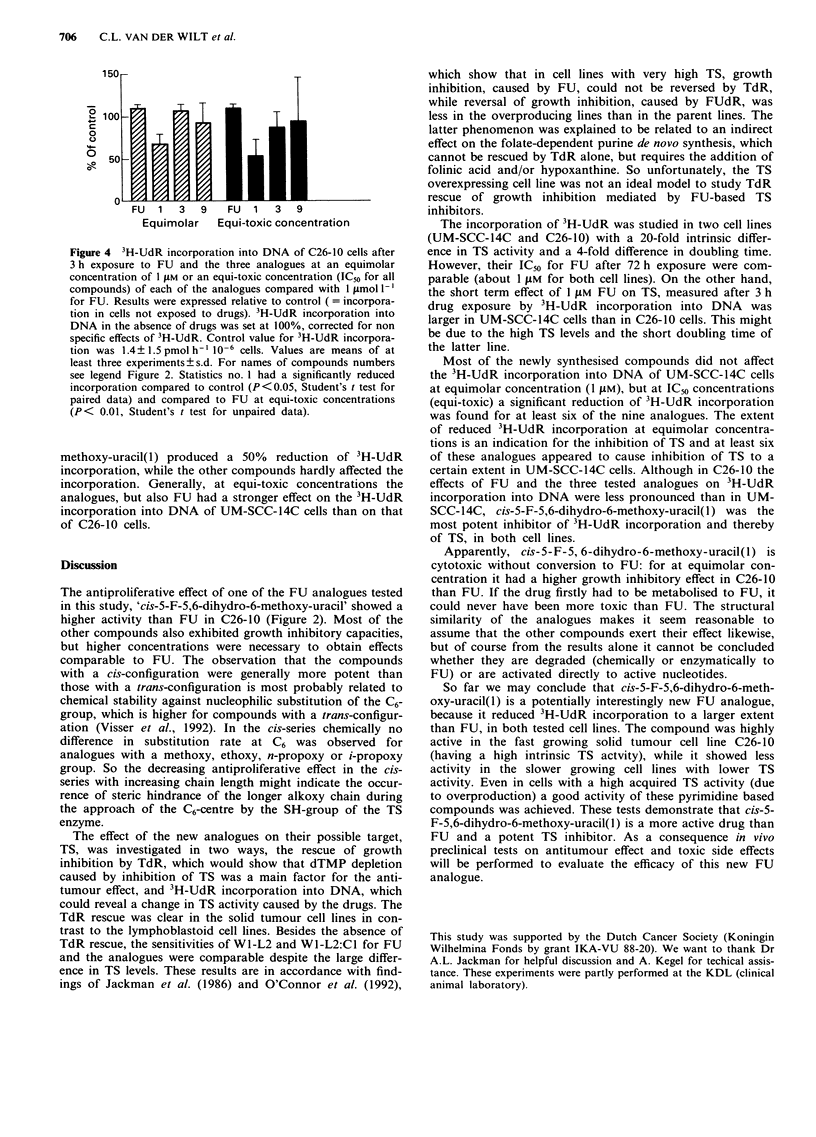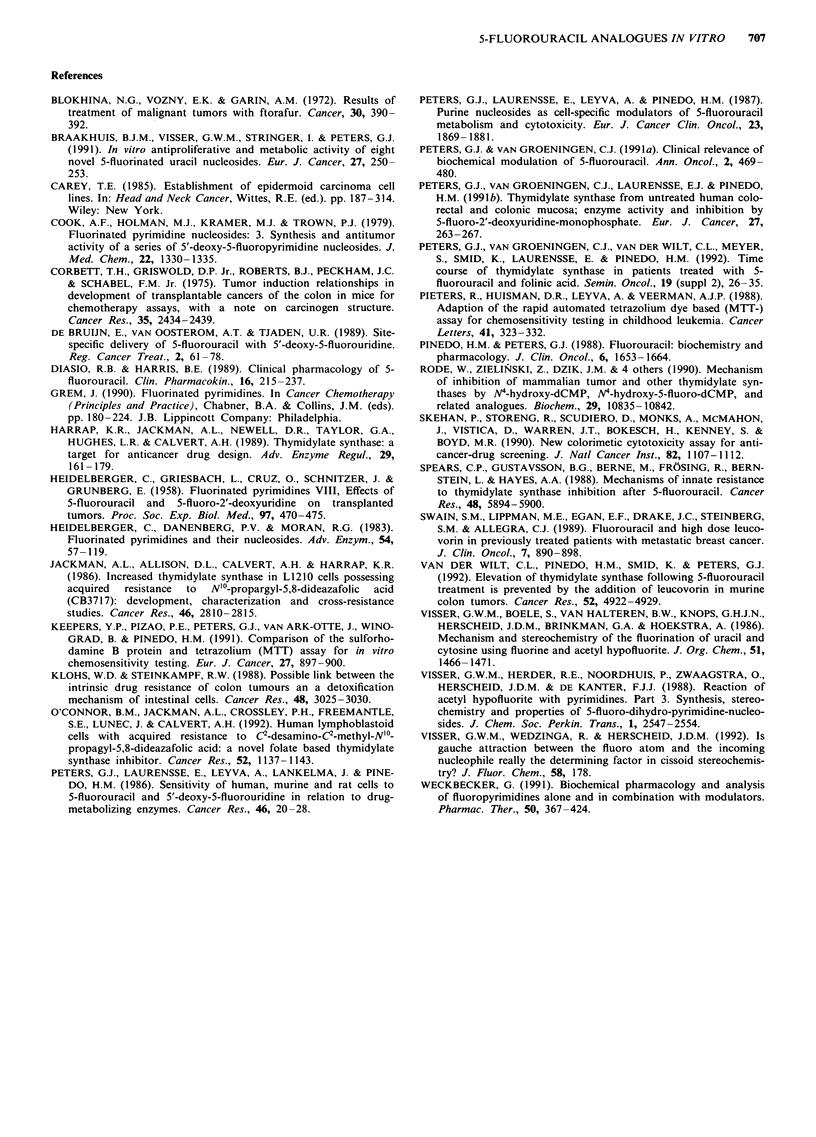# In vitro antitumour activity of cis- and trans-5-fluoro-5,6-dihydro-6-alkoxy-uracils; effects on thymidylate synthesis.

**DOI:** 10.1038/bjc.1993.413

**Published:** 1993-10

**Authors:** C. L. van der Wilt, G. W. Visser, B. J. Braakhuis, R. Wedzinga, P. Noordhuis, K. Smid, G. J. Peters

**Affiliations:** Department of Oncology, Free University Hospital, Amsterdam, Netherlands.

## Abstract

A class of new 5-fluorouracil (FU) analogues, the 5-fluoro-5,6-dihydro-6- alkoxy-uracils was synthesised with a modification at the 6-position of the pyrimidine ring. At this position the analogues have a hydroxy or alkoxy group of different chain lengths either in the cis- or trans-configuration. The antiproliferative effect of these compounds was tested on five cell lines of different origin. Generally, the analogues with a cis-configuration had a higher activity than those with a trans-configuration. The growth inhibitory effect of the compounds decreased with increasing alkoxy chain length, but the compound with a hydroxy group had the lowest growth inhibitory effect. One analogue, cis-5-F-5,6-dihydro-6-methoxy-uracil had a higher antiproliferative effect than FU in one of the cell lines. Effects on thymidylate synthase (TS), the possible target of these analogues, were evaluated by thymidine rescue of growth inhibition and incorporation of tritiated deoxyuridine (3H-UdR) into DNA. In solid tumour cell lines addition of TdR reversed the antiproliferative effect. Inhibition of TS in intact cells was determined by measuring 3H-UdR incorporation in two cell lines. The effect of cis-5-F-5,6-dihydro-6-methoxy-uracil on incorporation of 3H-UdR was 2- to 5-fold stronger than that of FU in both cell lines. All other compounds produced a higher 3H-UdR incorporation than FU both at equimolar and equi-toxic concentration. Concluding from these results we regard cis-5-F-5,6-dihydro-6-methoxy-uracil as the most promising FU analogue of this series, because of its higher antiproliferative activity than FU and marked inhibition of TS in intact cells.


Br. J. Cancer (1993), 68, 702-707 © Macmillan Press Ltd., 1993

### *In vitro* antitumour activity of *cis*- and *trans*-5-fluoro-5,6-dihydro-6-alkoxy-uracils; effects on thymidylate synthesis

C.L. van der Wilt[1], G.W.M. Visser[2], B.J.M. Braakhuis[3], R. Wedzinga[2], P. Noordhuis[1], K. Smid[1] & G.J. Peters[1]

[1]*Department of Oncology,* [2]*Radio-Nuclide-Centre, and* [3]*Department of Otolaryngology, Free University Hospital, PO Box 7057, 1007 MB Amsterdam, Netherlands.*

**Summary** A class of new 5-fluorouracil (FU) analogues, the 5-fluoro-5,6-dihydro-6-alkoxy-uracils was synthesised with a modification at the 6-position of the pyrimidine ring. At this position the analogues have a hydroxy or alkoxy group of different chain lengths either in the *cis*- or *trans*-configuration. The antiproliferative effect of these compounds was tested on five cell lines of different origin. Generally, the analogues with a *cis*-configuration had a higher activity than those with a *trans*-configuration. The growth inhibitory effect of the compounds decreased with increasing alkoxy chain length, but the compound with a hydroxy group had the lowest growth inhibitory effect. One analogue, *cis*-5-F-5,6-dihydro-6-methoxy-uracil had a higher antiproliferative effect than FU in one of the cell lines. Effects on thymidylate synthase (TS), the possible target of these analogues, were evaluated by thymidine rescue of growth inhibition and incorporation of tritiated deoxyuridine ($^3$H-UdR) into DNA. In solid tumour cell lines addition of TdR reversed the antiproliferative effect. Inhibition of TS in intact cells was determined by measuring $^3$H-UdR incorporation in two cell lines. The effect of *cis*-5-F-5,6-dihydro-6-methoxy-uracil on incorporation of $^3$H-UdR was 2- to 5-fold stronger than that of FU in both cell lines. All other compounds produced a higher $^3$H-UdR incorporation than FU both at equimolar and equi-toxic concentration. Concluding from these results we regard *cis*-5-F-5,6-dihydro-6-methoxy-uracil as the most promising FU analogue of this series, because of its higher antiproliferative activity than FU and marked inhibition of TS in intact cells.

Since its introduction as an antineoplastic agent 5-fluorouracil (FU) has been used for the treatment of a wide spectrum of solid tumours. However, objective response rates are moderate, being lower than 20% in controlled randomised trials of advanced colorectal cancer treatment and cures are rarely achieved (Weckbecker, 1991; Peters & Van Groeningen, 1991a). Several approaches have been used to increase this therapeutic index, such as combination with other anticancer drugs, biochemical modulation of the FU metabolism (Peters & Van Groeningen, 1991a) and synthesis of more potent analogues (Heidelberger *et al.*, 1983). All these studies are based on thorough research on the mechanism of action of FU, that has been performed during the last three decades (Pinedo & Peters, 1988; Diasio & Harris, 1989; Weckbecker, 1991).

The mechanism of action of FU is rather complicated and involves conversion to nucleotides. One of the most important nucleotides for antitumour activity is 5-fluoro-2′-deoxyuridine-5′-monophosphate (FdUMP), which blocks DNA synthesis through the inhibition of thymidylate synthase (TS, E.C.2.1.1.45), by the formation of a stable ternary complex with FdUMP and the cofactor 5,10-methylenetetrahydrofolate. Other nucleotides, that play a role in FU activity are 5-fluorouridine-5′-triphosphate (FUTP) and 5-fluoro-2′-deoxyuridine-5′-triphosphate (FdUTP). Incorporation of FUTP into RNA has been associated with both antitumour effect and gastrointestinal/myeloid toxicity. Whether incorporation of FdUTP into DNA, leading to DNA strand breaks and DNA fragmentation, really contributes to the antitumour effect could not be established until now (Pinedo & Peters, 1988). Supported by findings that a low inhibition of TS in tumours of patients treated with FU may be related to poor prognosis (Spears *et al.*, 1988), and that an enhanced antitumour effect seems to be related to a better inhibition of TS (Swain *et al.*, 1989; Peters *et al.*, 1991*b*, 1992), TS became an important target. Further research on TS and its inhibitors, included biochemical modulation of FU therapy with the folate cofactor precursor, folinic acid leading to an enhanced TS inhibition and an increase in the response (Swain *et al.*, 1989). This has promoted the development of potent analogues of the folate cofactor (Harrap *et al.*, 1989). Research concentrated to a lesser extent on analogues of FdUMP (Rode *et al.*, 1990) or nucleotides of FU analogues (Heidelberger *et al.*, 1983).

The early studies of FU derivatives were focused on the direction of FU precursors or prodrugs. These compounds are supposed to generate their activity by a more selective conversion to FU and/or its metabolites in tumour tissues. Examples of this group are floxuridine (5-fluoro-2′-deoxyuridine) (Heidelberger *et al.*, 1958), ftorafur ($N_1$-tetrahydrofuran-2-yl-5-fluorouracil) (Blokhina *et al.*, 1972) and doxifluridine (5′-deoxy-5-fluorouridine) (Cook *et al.*, 1979). These compounds did not show a clear therapeutic advantage over FU in systemic application (Weckbecker, 1991; Grem, 1990), although for some compounds the most optimal administration schedule has not (yet) been established (De Bruijn *et al.*, 1989).

Recently we described the synthesis and *in vitro* antitumour activity of a series of 5-fluorinated nucleosides (Visser *et al.*, 1988; Braakhuis *et al.*, 1991). In order to obtain more potent drugs in the class of fluorinated pyrimidines, we therefore synthesised a new series of compounds based on the structure of FU. The new analogues have a modification at the 6-position of the pyrimidine ring. The 5-F-5,6-dihydro-6-OR-uracil compounds have a R substitution of an hydroxy or alkoxy group of various chain length either in the *cis*- or *trans*-configuration (Figure 1). The rationale to develop this new series was to have an analogue that, due to the absence of a 5,6-double bound, is not directly a target for dihydropyrimidine dehydrogenase (DPD). DPD is an important catabolic enzyme, which inactivates a large part (up to 90%) of a FU dose *in vivo*, before it can reach the tumour (Diasio & Harris, 1989). Chemically the group at the 6-position of the analogue appeared to be susceptible to substituted, so theoretically in cells this could be substituted by the SH-group of TS. In addition, apart from the fact that the group at the 6-position creates a potentially interesting difference in lipophilicity, chemically the *trans* compound was found to be more stable than its corresponding *cis* compound. Finally the $N^1$H-group is still free and no problem with conversion to nucleosides and subsequently to nucleotides should theoretically occur. This implies that these compounds are no FU prodrugs.

This study describes the antiproliferative effect *in vitro* of

Correspondence: G.J. Peters.
Received 23 December 1992; and in revised form 28 April 1993.

**Figure 1** Schematic presentation of the synthesis and basic structure of 5-F-5,6-dihydro-6-alkoxy-uracil.

these 5-fluoro-5,6-dihydro-6-alkoxy-uracil adducts. Each compound has also been tested for its capacity to inhibit TS, the possible target for antitumour effect.

### Materials and methods

### *Synthesis*

The basic structure of 5-F-5,6-dihydro-6-alkoxy-uracil and the synthetic route are shown in Figure 1. The detailed synthesis of the 5-F-5,6-dihydro-6-alkoxy-uracil compounds, their conformation and their chemical properties will be described elsewhere, together with analysis and further characteristics. In short, the 5-F-5,6-dihydro-6-OAc-uracil adduct is formed from the reaction of gaseous AcOF with uracil in acetic acid (Visser *et al.*, 1986). Under acidic conditions ($H_2SO_4$) reaction of the 5-F,6-OAc adduct with ROH resulted within 15 min at 80°C in predominantly the corresponding *cis*-5-F-5,6-dihydro-6-alkoxy-uracil compounds; prolonged heating increased the amount of the *trans* compound resulting in a *cis*/*trans* ratio of about two. After addition of an aqueous $K_2CO_3$ solution until pH = 3, the solvent was roto-evaporated and the *cis*/*trans* compounds were separated by column chromatography (Lobar Lichoprep. Rp-8, 40–63 μm (Merck, Amsterdam, The Netherlands); eluent: ultrapure water). After collection, the water was removed by freeze-drying. The *cis*/*trans* stereochemistry of each adduct was established by $^1H$ NMR measurement of the $J_{5F,6H}$ coupling constant (*cis* isomers: $J_{5F,6H}$ 2.0–2.3 Hz; *trans* isomers $J_{5F,6H}$ 7.1–7.4 Hz). Due to anomeric effects (Visser *et al.*, 1986), all isomers have the preferred conformation with the OR-group in the axial position.

### *Drugs*

FU was obtained from Sigma (St Louis, MO, USA). All drugs were dissolved in sterile 0.9% NaCl and stored at −20°C at $10^{-2}$ mol $l^{-1}$ stock solution. Under these conditions all compounds were stable for several months.

### *Cell lines*

Two human cancer cell lines were used, UM-SCC-11B (doubling time 28 h), a moderately differentiated squamous cell carcinoma of the larynx and UM-SCC-14C (doubling time 26 h), a poorly differentiated squamous cell carcinoma of the oral cavity. Both cell lines were obtained from Dr T.E. Carey, University of Michigan, Ann Arbor, USA (Carey, 1985). A third cell line C26-10 (doubling time 18 h), was a gift from Dr Klohs (Klohs & Steinkampf, 1988) and was originally derived from a undifferentiated murine colon carcinoma (Corbett *et al.*, 1975). The two lymphoblastoid cell lines W1-L2 and W1-L2:C1 (a gift from Dr A.L. Jackman) have been added to the panel, because the W1-L2:C1 has an acquired resistance against a folate-based TS inhibitor, which resulted in a 200-fold overexpression of the TS protein and TS activity compared to the parental W1-L2 (O'Connor *et al.*, 1992).

The lymphoblastoid cell lines were grown in RPMI 1640 (Flow Laboratories, Irvine, Scotland). The other cells were routinely grown in Dulbecco's modified Eagle's medium (DMEM, Flow). DMEM and RPMI medium were supplemented with 10% heat inactivated foetal calf serum (FCS) (Gibco, New York, USA), and 1 mM L-glutamine. During experiments 100 U $ml^{-1}$ penicillin and 100 μg $ml^{-1}$ streptomycin were added to the culture medium.

### *Growth inhibition tests*

Cells from routine, subconfluent cultures were transferred to 96-well flat-bottom plates (Greiner Labortechnik, Solingen, Germany) (C26-10, UM-SCC-11B and UM-SCC-14C) or round-bottom plates (Greiner) (W1-L2 and W1-L2:C1). For the UM-SCC-11B and UM-SCC-14C cell density was 1500 cells/well in 150 μl medium. After a lag-phase of 72 h, 50 μl of drug containing medium was added. C26-10 cells were plated at a density of 1000 cells/well in 100 μl and after 24 h 100 μl drug containing medium was added. W1-L2 and W1-L2:C1 were seeded at a density of 10,000 cells/well in 50 μl and 1 h later 50 μl drug containing medium was added. For the thymidine (TdR) rescue experiments 10 μM (final concentration) was added simultaneously with FU or one of the analogues. Drug exposure time was 72 h for all cell lines. The drugs were tested in triplicate at concentrations ranging from $10^{-4}$ to $10^{-8}$ M. Growth inhibitory effects were evaluated with the standard sulforhodamine B (SRB) assay (Skehan *et al.*, 1990; Keepers *et al.*, 1991) for C26-10, UM-SCC-11B and UM-SCC-14C and with the MTT assay (Pieters *et al.*, 1988) for W1-L2 and W1-L2:C1. The $IC_{50}$ was the concentration that corresponded to half-maximal growth of the control based on the difference of optical density values at the start and the end of drug exposure. The $IC_{50}$ concentrations were determined from at least three separate experiments.

### *Thymidylate synthase assay*

The activity of the enzyme TS was measured with two assays, a ligand binding assay with [6-$^3$H]-FdUMP (Moravek, Brea, CA, USA; 20 Ci $mmol^{-1}$), with which the free FdUMP binding sites of TS can be determined. The catalytic activity of the enzyme was determined by measurement of the conversion of [5-$^3$H]-dUMP into dTMP and $^3H_2O$ at two substrate concentrations of dUMP, 1 and 10 μM. In this assay we evaluated the inhibitory effect of 10 nM FdUMP. Both the FdUMP binding assay and the $^3$H-release assay were performed on 7000 *g* supernatants of sonicated cell extracts of $20 \times 10^6$ cells. Appropriate controls for determination of linearity with time and protein were included. Details of the assay procedure have been described extensively elsewhere (Peters *et al.*, 1986; Van der Wilt *et al.*, 1992).

*Incorporation of [6-³H]-deoxyuridine into DNA*

The incorporation of [6-³H]-deoxyuridine (³H-UdR) (Amersham International, Buckinghamshire, UK; specific activity 17 Ci mmol$^{-1}$) into DNA was measured in two of the solid tumour cell lines with the lowest and highest TS activity, UM-SCC-14C and C26-10, respectively. Two concentrations of each 5-F-5,6-dihydro-6-alkoxy-uracil compound were tested and compared to the effect of FU. All compounds were tested at the concentration causing 50% growth inhibition (equi-toxic) and at the equimolar concentration of 1 μM. The assay was performed as described previously (Peters *et al.*, 1987; Braakhuis *et al.*, 1991). Cells ($10^5$/well in 100 μl) were seeded in 96-well filtration plates coated with a hydrophilic PVDF filter with 0.22 μm pore size (Millipore Corporation, Bedford, MA, USA). After 24 h 100 μl drug containing medium was added and the cells were incubated with the drug for 1 h. Then 10 μl 10 μM ³H-UdR (0.5 μCi; specific activity 4.5 Ci mmol$^{-1}$) was added. After another 2 h incubation the plate was put on a Millipore vacuum holder and the medium was filtered through the membrane of the wells using a vacuum pump. Next, the cells were precipitated by addition of 200 μl ice-cold 8% trichloroacetic acid (TCA), followed by three washes with 8% ice-cold TCA, four washes with water and subsequently with 70% ethanol (4 ×). After drying by cold air the filters were collected with a multiple punch assembly (Millipore), and 500 μl 2 M NaOH was added to solubilise the precipitated nucleic acids. Finally radioactivity was counted after addition of 5 ml Optiphase III (Hisafe) liquid scintillation fluid (LKB, Woerden, The Netherlands). Values were corrected for non-specific binding of ³H-UdR.

### Results

*Growth inhibition*

The antiproliferative effect of the 5-F-5,6-dihydro-6-alkoxy-uracil compounds compared to FU in the three solid tumour cell lines has been summarised in Figure 2. All compounds were tested on UM-SCC-14C and UM-SCC-11B, because of previous testing of fluoropyrimidine analogues in these cells (Braakhuis *et al.*, 1991). Besides this, because of different sensitivity for FU, these cell lines form a good model to get an insight in the spectrum of activity of the new drugs.

FU appeared to be the most active drug in these two cell lines. In general the analogues with a *cis*-configuration (the odd numbers 1–7) had a higher growth inhibitory effect than the analogues with a *trans*-configuration (the even numbers). The antiproliferative effect of the compounds decreased with increasing chain length of the alkoxy substitution, which was illustrated by the step-like pattern of compounds number 2, 4, 6 and 8 (Figure 2). *Cis*-5-F-5,6-dihydro-6-hydroxy-uracil(9) showed a very poor growth inhibitory effect in these two cells lines.

For C26-10 (Figure 2), the third solid tumour cell line in the panel only *cis*-5-F-5,6-dihydro-6-methoxy-uracil(1) and *cis*-5-F-5,6-dihydro-6-ethoxy-uracil(3), the most promising active compounds from the previous growth inhibition tests, were tested. *Cis*-5-F-5,6-dihydro-6-hydroxy-uracil(9) was included as a negative control, in view of its very low activity in the other two cell lines. Interestingly, the analogue *cis*-5-F-5,6-dihydro-6-methoxy-uracil(1) had a striking effect on C26-10, with a better growth inhibition than FU at equimolar concentration.

Additionally TdR rescue experiments were performed to evaluate whether growth inhibitory effects mediated by TS, could be circumvented by the addition of 10 μM TdR. For these experiments we included the lymphoblastoid cell lines in the panel. W1-L2:C1, which has a very high TS expression, might be a good cell line to study TS related growth inhibition. Results on the TdR rescue are summarised in Table I. The dose modifying factor ($IC_{50}$ of drug + TdR/$IC_{50}$ of drug alone) varied for each compound and the cell line. Generally TdR rescued growth inhibition mediated by all compounds in UM-SCC-11B, UM-SCC-14C and C26-10 (except compound no. 9), although not always the level of significance was achieved. The lymphoblastoid cell lines did not differ very much with respect to their sensitivity for FU and the analogues (Figure 2) and TdR rescue was very limited in these cell lines (Table I).

*Thymidylate synthase activity*

The activity of TS was measured firstly as the number of binding sites to TS for FdUMP, the active nucleotide of FU and secondly as the catalytic activity to convert its natural substrate dUMP to dTMP. The results of both assays, measured in cells that were not exposed to FU, were very different for each cell line (Table II). The FdUMP binding capacity of the fast growing C26-10 cell lines was about 4-fold higher than that of UM-SCC-11B and about 20-fold higher than that of UM-SCC-14C. For the catalytic activity

**Figure 2** *In vitro* growth inhibitory effects of the new FU analogues expressed as relative $IC_{50}$ as compared to the $IC_{50}$ of FU (1.1 ± 0.4 μM for UM-SCC-14C, 5.6 ± 1.2 μM for UM-SCC-11B, 0.63 ± 0.19 μM for C26-10, 5.7 ± 1.2 μM for W1-L2 and 4.8 ± 1.1 μM for W1-L2:C1). Values are means of at least three experiments ± s.d. 1 = *cis*-5-F-5,6-dihydro-6-methoxy-uracil; 2 = *trans*-5-F-5,dihydro-6-methoxy-uracil; 3 = *cis*-5-F-5,6-dihydro-6-ethoxy-uracil; 4 = *trans*-5-F-5,6-dihydro-6-ethoxy-uracil; 5 = *cis*-5-F-5,6-dihydro-6-*n*-propoxy-uracil; 6 = *trans*-5-F-5,6-dihydro-6-*n*-propoxy-uracil; 7 = *cis*-5-F-5,6-dihydro-6-*i*-propoxy-uracil; 8 = *trans*-5-F-5,6-dihydro-6-*i*-propoxy-uracil; 9 = *cis*-5-F-5,6-dihydro-6-hydroxy-uracil. Statistics (Student's *t*-test for unpaired data); in UM-SCC-11B the following compounds had a significantly smaller antiproliferative effect than FU, no. 5, $P < 0.02$; no. 6, $P < 0.05$; no. 8, $P < 0.01$ and no. 9, $P < 0.001$. In UM-SCC-14C FU was significantly more active than the *trans*-5-F-5,6-dihydro-6-alkoxy-uracil compounds (no. 2, 4, 6, $P < 0.05$; no. 8, $P < 0.001$; no. 9, $P < 0.01$). In C26-10 compound no. 1 was significantly more active than FU ($P < 0.001$), while the other two, compounds no. 3 and 9 were less active than FU ($P < 0.001$). In W1-L2 and W1-L2:C1 compound no. 9 was significantly less active than FU ($P < 0.001$).

**Table I** Thymidine rescue of antiproliferative effects

| | *UM-SCC-11B* | *UM-SCC-14C* | *C26-10* | *WI-L2* | *WI-L2:C1* |
|---|---|---|---|---|---|
| | | | *dose modifying factor*[a] | | |
| FU | 2.0±0.7 (4)[b] | 3.5±1.5 (3) | 1.5±0.4 (4)[b] | 0.9±0.1 (4) | 1.6±0.5 (4) |
| no. 1 | 2.1±1.2 (4) | 4.8±2.6 (4)[b] | 1.3±0.2 (5)[b] | 2.2±1.7 (4) | 0.9±0.2 (5) |
| no. 3 | 1.4±0.9 (4)[b] | 2.8±0.6 (3)[b] | 1.4±0.2 (5) | 1.0±0.3 (4) | 1.2±0.5 (5) |
| no. 9 | 2.2±1.3 (4) | 3.1±1.7 (3) | 1.1±0.3 (4) | 1.0 (1) | 1.0±0.0 (2) |

Values are means ± s.d. The TdR concentration was 10 μM. [a]Dose modifying factor $IC_{50}/IC_{50}$ of drug alone. [b]Significant rescue by addition of TdR ($P < 0.05$, paired Student's *t* test) (see Figure 2 for compound number and name).

**Table II** FdUMP binding and catalytic activity of thymidylate synthase

| | *UM-SCC-14C* | *UM-SCC-11B* | *C26-10* |
|---|---|---|---|
| *FdUMP binding* | | *(fmol $10^{-6}$ cells)* | |
| | 23±9 | 122±63 | 522±103 |
| *Catalytic activity* | | *(pmol $h^{-1}$ $10^{-6}$ cells)* | |
| at 1 μM dUMP | 15±13 | 98±59 | 231±110 |
| at 10 μM dUMP | 57±37 | 368±225 | 1052±244 |
| inhibition by 10 nM FdUMP | | *(pmol $h^{-1}$ $10^{-6}$ cells) (% of control)* | |
| at 1 μM dUMP | 4.8±2.5 (32%) | 19±13 (19%) | 65±37 (28%) |
| at 10 μM dUMP | 21±17 (36%) | 141±98 (38%) | 449±123 (42%) |

Values are means ± s.d., $n = 3$. Protein content of the cells was 6370, 7260 and 6970 μg protein $10^{-6}$ cells for UM-SCC-14C, UM-SCC-11B and C26-10, respectively.

differences of comparable magnitude were observed between the cell lines. Finally the addition of FdUMP in the $^3$H-release assay inhibited the TS catalytic activity for about 70% in all three cell lines at both substrate concentrations. Data on catalytic activity of WI-L2 and WI-L2:C1 were published by O'Connor *et al.* (1992) (13,400 and 2,410,000 pmol $h^{-1}$ $mg^{-1}$ protein, respectively).

### *$^3$H-UdR incorporation*

In order to determine whether these compounds exerted their antiproliferative effect through the inhibition of TS, we measured the incorporation of $^3$H-UdR into DNA. [6-$^3$H]-UdR is converted into [6-$^3$H]-dUMP, followed by a TS catalysed formation of [6-$^3$H]-dTMP, one of the precursors for DNA synthesis. Inhibition of TS reduced the amount of $^3$H incorporated into DNA of both UM-SCC-14C cells (Figure 3) and C26-10 cells (Figure 4) with a relatively low and high TS activity, respectively.

The analogues were tested at a concentration of 1 μM. This is equimolar to the $IC_{50}$ of FU after 72 h drug exposure in both cell lines. Although, the drug exposure time in this assay was only 3 h, exposure to 1 μM FU resulted already in about 50% inhibition of $^3$H-UdR incorporation, because inhibition of TS is a fast event and precedes growth inhibition. Thus this method can be used as a fast screening test for inhibitory effects on thymidylate synthesis. At the equimolar concentration only FU, *cis*-5-F-5,6-dihydro-6-methoxy-uracil(1) and *cis*-5-F-5,6-dihydro-6-*n*-propoxy-uracil(5) reduced $^3$H-UdR incorporation in UM-SCC-14C (Figure 3a). At equi-toxic concentration (the $IC_{50}$ concentration of each compound after 72 h drug exposure) *cis*-5-F-5,6-dihydro-6-*n*-propoxy-uracil(5) and *cis*-5-F-5,6-dihydro-6-hydroxy-uracil(9) produced a reduction of $^3$H-UdR incorporation into the DNA of UM-SCC-14C cells comparable to that of FU (Figure 3b). Three hours incubation of UM-SCC-14C cells with *cis*-5-F-5,6-dihydro-6-methoxy-uracil(1) resulted in an even better reduction of $^3$H-UdR incorporation than incubation with FU, during the same period.

In C26-10 cells exposure to *cis*-5-F-5,6-dihydro-6-methoxy-uracil(1) at equimolar concentration resulted in an incorporation significantly lower than control ($P < 0.05$) (Figure 4a). The other two drugs and also FU did not reduce the $^3$H-UdR incorporation at this concentration and short exposure time. At equi-toxic concentration only *cis*-5-F-5,6-dihydro-6-

**Figure 3** $^3$H-UdR incorporation into DNA of UM-SCC-14C cells after 3 h exposure to FU and the various analogues at an equimolar concentration of 1 μM **a**, or an equitoxic concentration ($IC_{50}$ for all compounds) of each of the analogues with 1 μM for FU **b**. Results were expressed relative to control. This is $^3$H-UdR incorporation into DNA in the absence of drugs, which was set at 100%, corrected for non specific effects of $^3$H-UdR. Control value for $^3$H-UdR incorporation was 2.7±1.8 pmol $h^{-1}$ $10^{-6}$ cells. Values are means of at least three experiments ± s.d. For names of compound numbers see legend Figure 2. Statistics **a**, FU and analogues 1 and 5 had a significantly reduced incorporation compared to control ($P < 0.05$, Student's *t* test for paired data). **b**, Compounds no. 2, 3, 6, 7 and 8 had a significantly higher incorporation than FU ($P < 0.05$, Student's *t* test for unpaired data).

**Figure 4** $^3$H-UdR incorporation into DNA of C26-10 cells after 3 h exposure to FU and the three analogues at an equimolar concentration of 1 $\mu$M or an equi-toxic concentration ($IC_{50}$ for all compounds) of each of the analogues compared with 1 $\mu mol\ l^{-1}$ for FU. Results were expressed relative to control ( = incorporation in cells not exposed to drugs). $^3$H-UdR incorporation into DNA in the absence of drugs was set at 100%, corrected for non specific effects of $^3$H-UdR. Control value for $^3$H-UdR incorporation was $1.4 \pm 1.5$ pmol $h^{-1}$ $10^{-6}$ cells. Values are means of at least three experiments ± s.d. For names of compounds numbers see legend Figure 2. Statistics no. 1 had a significantly reduced incorporation compared to control ($P < 0.05$, Student's $t$ test for paired data) and compared to FU at equi-toxic concentrations ($P < 0.01$, Student's $t$ test for unpaired data).

methoxy-uracil(1) produced a 50% reduction of $^3$H-UdR incorporation, while the other compounds hardly affected the incorporation. Generally, at equi-toxic concentrations the analogues, but also FU had a stronger effect on the $^3$H-UdR incorporation into DNA of UM-SCC-14C cells than on that of C26-10 cells.

### Discussion

The antiproliferative effect of one of the FU analogues tested in this study, '*cis*-5-F-5,6-dihydro-6-methoxy-uracil' showed a higher activity than FU in C26-10 (Figure 2). Most of the other compounds also exhibited growth inhibitory capacities, but higher concentrations were necessary to obtain effects comparable to FU. The observation that the compounds with a *cis*-configuration were generally more potent than those with a *trans*-configuration is most probably related to chemical stability against nucleophilic substitution of the $C_6$-group, which is higher for compounds with a *trans*-configuration (Visser *et al.*, 1992). In the *cis*-series chemically no difference in substitution rate at $C_6$ was observed for analogues with a methoxy, ethoxy, *n*-propoxy or *i*-propoxy group. So the decreasing antiproliferative effect in the *cis*-series with increasing chain length might indicate the occurrence of steric hindrance of the longer alkoxy chain during the approach of the $C_6$-centre by the SH-group of the TS enzyme.

The effect of the new analogues on their possible target, TS, was investigated in two ways, the rescue of growth inhibition by TdR, which would show that dTMP depletion caused by inhibition of TS was a main factor for the anti-tumour effect, and $^3$H-UdR incorporation into DNA, which could reveal a change in TS activity caused by the drugs. The TdR rescue was clear in the solid tumour cell lines in contrast to the lymphoblastoid cell lines. Besides the absence of TdR rescue, the sensitivities of W1-L2 and W1-L2:C1 for FU and the analogues were comparable despite the large difference in TS levels. These results are in accordance with findings of Jackman *et al.* (1986) and O'Connor *et al.* (1992), which show that in cell lines with very high TS, growth inhibition, caused by FU, could not be reversed by TdR, while reversal of growth inhibition, caused by FUdR, was less in the overproducing lines than in the parent lines. The latter phenomenon was explained to be related to an indirect effect on the folate-dependent purine *de novo* synthesis, which cannot be rescued by TdR alone, but requires the addition of folinic acid and/or hypoxanthine. So unfortunately, the TS overexpressing cell line was not an ideal model to study TdR rescue of growth inhibition mediated by FU-based TS inhibitors.

The incorporation of $^3$H-UdR was studied in two cell lines (UM-SCC-14C and C26-10) with a 20-fold intrinsic difference in TS activity and a 4-fold difference in doubling time. However, their $IC_{50}$ for FU after 72 h exposure were comparable (about 1 $\mu$M for both cell lines). On the other hand, the short term effect of 1 $\mu$M FU on TS, measured after 3 h drug exposure by $^3$H-UdR incorporation into DNA was larger in UM-SCC-14C cells than in C26-10 cells. This might be due to the high TS levels and the short doubling time of the latter line.

Most of the newly synthesised compounds did not affect the $^3$H-UdR incorporation into DNA of UM-SCC-14C cells at equimolar concentration (1 $\mu$M), but at $IC_{50}$ concentrations (equi-toxic) a significant reduction of $^3$H-UdR incorporation was found for at least six of the nine analogues. The extent of reduced $^3$H-UdR incorporation at equimolar concentrations is an indication for the inhibition of TS and at least six of these analogues appeared to cause inhibition of TS to a certain extent in UM-SCC-14C cells. Although in C26-10 the effects of FU and the three tested analogues on $^3$H-UdR incorporation into DNA were less pronounced than in UM-SCC-14C, *cis*-5-F-5,6-dihydro-6-methoxy-uracil(1) was the most potent inhibitor of $^3$H-UdR incorporation and thereby of TS, in both cell lines.

Apparently, *cis*-5-F-5, 6-dihydro-6-methoxy-uracil(1) is cytotoxic without conversion to FU: for at equimolar concentration it had a higher growth inhibitory effect in C26-10 than FU. If the drug firstly had to be metabolised to FU, it could never have been more toxic than FU. The structural similarity of the analogues makes it seem reasonable to assume that the other compounds exert their effect likewise, but of course from the results alone it cannot be concluded whether they are degraded (chemically or enzymatically to FU) or are activated directly to active nucleotides.

So far we may conclude that *cis*-5-F-5,6-dihydro-6-methoxy-uracil(1) is a potentially interestingly new FU analogue, because it reduced $^3$H-UdR incorporation to a larger extent than FU, in both tested cell lines. The compound was highly active in the fast growing solid tumour cell line C26-10 (having a high intrinsic TS actvity), while it showed less activity in the slower growing cell lines with lower TS activity. Even in cells with a high acquired TS activity (due to overproduction) a good activity of these pyrimidine based compounds was achieved. These tests demonstrate that *cis*-5-F-5,6-dihydro-6-methoxy-uracil(1) is a more active drug than FU and a potent TS inhibitor. As a consequence *in vivo* preclinical tests on antitumour effect and toxic side effects will be performed to evaluate the efficacy of this new FU analogue.

This study was supported by the Dutch Cancer Society (Koningin Wilhelmina Fonds by grant IKA-VU 88-20). We want to thank Dr A.L. Jackman for helpful discussion and A. Kegel for techical assistance. These experiments were partly performed at the KDL (clinical animal laboratory).

### References

BLOKHINA, N.G., VOZNY, E.K. & GARIN, A.M. (1972). Results of treatment of malignant tumors with ftorafur. *Cancer*, **30**, 390–392.

BRAAKHUIS, B.J.M., VISSER, G.W.M., STRINGER, I. & PETERS, G.J. (1991). *In vitro* antiproliferative and metabolic activity of eight novel 5-fluorinated uracil nucleosides. *Eur. J. Cancer*, **27**, 250–253.

CAREY, T.E. (1985). Establishment of epidermoid carcinoma cell lines. In: *Head and Neck Cancer*, Wittes, R.E. (ed.). pp. 187–314. Wiley: New York.

COOK, A.F., HOLMAN, M.J., KRAMER, M.J. & TROWN, P.J. (1979). Fluorinated pyrimidine nucleosides: 3. Synthesis and antitumor activity of a series of 5′-deoxy-5-fluoropyrimidine nucleosides. *J. Med. Chem.*, **22**, 1330–1335.

CORBETT, T.H., GRISWOLD, D.P. Jr., ROBERTS, B.J., PECKHAM, J.C. & SCHABEL, F.M. Jr. (1975). Tumor induction relationships in development of transplantable cancers of the colon in mice for chemotherapy assays, with a note on carcinogen structure. *Cancer Res.*, **35**, 2434–2439.

DE BRUIJN, E., VAN OOSTEROM, A.T. & TJADEN, U.R. (1989). Site-specific delivery of 5-fluorouracil with 5′-deoxy-5-fluorouridine. *Reg. Cancer Treat.*, **2**, 61–78.

DIASIO, R.B. & HARRIS, B.E. (1989). Clinical pharmacology of 5-fluorouracil. *Clin. Pharmacokin.*, **16**, 215–237.

GREM, J. (1990). Fluorinated pyrimidines. In *Cancer Chemotherapy (Principles and Practice)*, Chabner, B.A. & Collins, J.M. (eds). pp. 180–224. J.B. Lippincott Company: Philadelphia.

HARRAP, K.R., JACKMAN, A.L., NEWELL, D.R., TAYLOR, G.A., HUGHES, L.R. & CALVERT, A.H. (1989). Thymidylate synthase: a target for anticancer drug design. *Adv. Enzyme Regul.*, **29**, 161–179.

HEIDELBERGER, C., GRIESBACH, L., CRUZ, O., SCHNITZER, J. & GRUNBERG, E. (1958). Fluorinated pyrimidines VIII, Effects of 5-fluorouracil and 5-fluoro-2′-deoxyuridine on transplanted tumors. *Proc. Soc. Exp. Biol. Med.*, **97**, 470–475.

HEIDELBERGER, C., DANENBERG, P.V. & MORAN, R.G. (1983). Fluorinated pyrimidines and their nucleosides. *Adv. Enzym.*, **54**, 57–119.

JACKMAN, A.L., ALLISON, D.L., CALVERT, A.H. & HARRAP, K.R. (1986). Increased thymidylate synthase in L1210 cells possessing acquired resistance to $N^{10}$-propargyl-5,8-dideazafolic acid (CB3717): development, characterization and cross-resistance studies. *Cancer Res.*, **46**, 2810–2815.

KEEPERS, Y.P., PIZAO, P.E., PETERS, G.J., VAN ARK-OTTE, J., WINOGRAD, B. & PINEDO, H.M. (1991). Comparison of the sulforhodamine B protein and tetrazolium (MTT) assay for *in vitro* chemosensitivity testing. *Eur. J. Cancer*, **27**, 897–900.

KLOHS, W.D. & STEINKAMPF, R.W. (1988). Possible link between the intrinsic drug resistance of colon tumours an a detoxification mechanism of intestinal cells. *Cancer Res.*, **48**, 3025–3030.

O'CONNOR, B.M., JACKMAN, A.L., CROSSLEY, P.H., FREEMANTLE, S.E., LUNEC, J. & CALVERT, A.H. (1992). Human lymphoblastoid cells with acquired resistance to $C^2$-desamino-$C^2$-methyl-$N^{10}$-propagyl-5,8-dideazafolic acid: a novel folate based thymidylate synthase inhibitor. *Cancer Res.*, **52**, 1137–1143.

PETERS, G.J., LAURENSSE, E., LEYVA, A., LANKELMA, J. & PINEDO, H.M. (1986). Sensitivity of human, murine and rat cells to 5-fluorouracil and 5′-deoxy-5-fluorouridine in relation to drug-metabolizing enzymes. *Cancer Res.*, **46**, 20–28.

PETERS, G.J., LAURENSSE, E., LEYVA, A. & PINEDO, H.M. (1987). Purine nucleosides as cell-specific modulators of 5-fluorouracil metabolism and cytotoxicity. *Eur. J. Cancer Clin. Oncol.*, **23**, 1869–1881.

PETERS, G.J. & VAN GROENINGEN, C.J. (1991*a*). Clinical relevance of biochemical modulation of 5-fluorouracil. *Ann. Oncol.*, **2**, 469–480.

PETERS, G.J., VAN GROENINGEN, C.J., LAURENSSE, E.J. & PINEDO, H.M. (1991*b*). Thymidylate synthase from untreated human colorectal and colonic mucosa; enzyme activity and inhibition by 5-fluoro-2′-deoxyuridine-monophosphate. *Eur. J. Cancer*, **27**, 263–267.

PETERS, G.J., VAN GROENINGEN, C.J., VAN DER WILT, C.L., MEYER, S., SMID, K., LAURENSSE, E. & PINEDO, H.M. (1992). Time course of thymidylate synthase in patients treated with 5-fluorouracil and folinic acid. *Semin. Oncol.*, **19** (suppl 2), 26–35.

PIETERS, R., HUISMAN, D.R., LEYVA, A. & VEERMAN, A.J.P. (1988). Adaption of the rapid automated tetrazolium dye based (MTT-) assay for chemosensitivity testing in childhood leukemia. *Cancer Letters*, **41**, 323–332.

PINEDO, H.M. & PETERS, G.J. (1988). Fluorouracil: biochemistry and pharmacology. *J. Clin. Oncol.*, **6**, 1653–1664.

RODE, W., ZIELIŃSKI, Z., DZIK, J.M. & 4 others (1990). Mechanism of inhibition of mammalian tumor and other thymidylate synthases by $N^4$-hydroxy-dCMP, $N^4$-hydroxy-5-fluoro-dCMP, and related analogues. *Biochem.*, **29**, 10835–10842.

SKEHAN, P., STORENG, R., SCUDIERO, D., MONKS, A., MCMAHON, J., VISTICA, D., WARREN, J.T., BOKESCH, H., KENNEY, S. & BOYD, M.R. (1990). New colorimetic cytotoxicity assay for anti-cancer-drug screening. *J. Natl Cancer Inst.*, **82**, 1107–1112.

SPEARS, C.P., GUSTAVSSON, B.G., BERNE, M., FRÖSING, R., BERNSTEIN, L. & HAYES, A.A. (1988). Mechanisms of innate resistance to thymidylate synthase inhibition after 5-fluorouracil. *Cancer Res.*, **48**, 5894–5900.

SWAIN, S.M., LIPPMAN, M.E., EGAN, E.F., DRAKE, J.C., STEINBERG, S.M. & ALLEGRA, C.J. (1989). Fluorouracil and high dose leucovorin in previously treated patients with metastatic breast cancer. *J. Clin. Oncol.*, **7**, 890–898.

VAN DER WILT, C.L., PINEDO, H.M., SMID, K. & PETERS, G.J. (1992). Elevation of thymidylate synthase following 5-fluorouracil treatment is prevented by the addition of leucovorin in murine colon tumors. *Cancer Res.*, **52**, 4922–4929.

VISSER, G.W.M., BOELE, S., VAN HALTEREN, B.W., KNOPS, G.H.J.N., HERSCHEID, J.D.M., BRINKMAN, G.A. & HOEKSTRA, A. (1986). Mechanism and stereochemistry of the fluorination of uracil and cytosine using fluorine and acetyl hypofluorite. *J. Org. Chem.*, **51**, 1466–1471.

VISSER, G.W.M., HERDER, R.E., NOORDHUIS, P., ZWAAGSTRA, O., HERSCHEID, J.D.M. & DE KANTER, F.J.J. (1988). Reaction of acetyl hypofluorite with pyrimidines. Part 3. Synthesis, stereochemistry and properties of 5-fluoro-dihydro-pyrimidine-nucleosides. *J. Chem. Soc. Perkin. Trans.*, **1**, 2547–2554.

VISSER, G.W.M., WEDZINGA, R. & HERSCHEID, J.D.M. (1992). Is gauche attraction between the fluoro atom and the incoming nucleophile really the determining factor in cissoid stereochemistry? *J. Fluor. Chem.*, **58**, 178.

WECKBECKER, G. (1991). Biochemical pharmacology and analysis of fluoropyrimidines alone and in combination with modulators. *Pharmac. Ther.*, **50**, 367–424.